# Crop‐to‐wild gene flow and its fitness consequences for a wild fruit tree: Towards a comprehensive conservation strategy of the wild apple in Europe

**DOI:** 10.1111/eva.12441

**Published:** 2016-12-26

**Authors:** Alice Feurtey, Amandine Cornille, Jacqui A. Shykoff, Alodie Snirc, Tatiana Giraud

**Affiliations:** ^1^ Ecologie Systématique EvolutionUniv. Paris‐SudCNRSAgroParisTechUniversité Paris‐SaclayOrsayFrance; ^2^ Department of Ecology and GeneticsEvolutionary Biology CentreScience for Life LaboratoryUppsala UniversityUppsalaSweden; ^3^ Adaptation to a Changing EnvironmentETH ZurichZurichSwitzerland; ^4^ Present address: Amandine Cornille, Institute of Integrative BiologyETH ZürichZürichSwitzerland

**Keywords:** agroforestry, agrosystems, crab apple, genetic swamping, introgression, orchards, pollination

## Abstract

Crop‐to‐wild gene flow can reduce the fitness and genetic integrity of wild species. *Malus sylvestris*, the European crab‐apple fruit tree in particular, is threatened by the disappearance of its habitat and by gene flow from its domesticated relative*, Malus domestica*. With the aims of evaluating threats for *M. sylvestris* and of formulating recommendations for its conservation, we studied here, using microsatellite markers and growth experiments: (i) hybridization rates in seeds and trees from a French forest and in seeds used for replanting crab apples in agrosystems and in forests, (ii) the impact of the level of *M. domestica* ancestry on individual tree fitness and (iii) pollen dispersal abilities in relation to crop‐to‐wild gene flow. We found substantial contemporary crop‐to‐wild gene flow in crab‐apple tree populations and superior fitness of hybrids compared to wild seeds and seedlings. Using paternity analyses, we showed that pollen dispersal could occur up to 4 km and decreased with tree density. The seed network furnishing the wild apple reintroduction agroforestry programmes was found to suffer from poor genetic diversity, introgressions and species misidentification. Overall, our findings indicate supported threats for the European wild apple steering us to provide precise recommendations for its conservation.

## 1 Introduction

Interspecific hybridization can have important evolutionary consequences, especially in plants, by fostering adaptation or on the contrary introducing ill‐suited alleles into populations. Gene flow between cultivated species and their wild relatives is of particular interest because it can be frequent (Ellstrand et al., 2013) and can impact the adaptive potential of both wild and domesticated species. Introgression from wild species into their cultivated relatives can contribute to variety improvement. For instance, hybridization between maize and the wild teosinte *Zea mays* spp. *mexicana* is likely to have contributed to crop resistance to the environmental conditions of the Mexican highlands (Hufford et al., 2013). On the other hand, hybridization between cultivated species and their wild relatives may have negative economical and ecological consequences. Hybrids growing in cultivated fields with their domesticated parents can reduce crop yield, for example in beets and carrots (Ellstrand et al., 2013). Hybridization from cultivated to wild populations can threaten the persistence of wild taxa, through either (i) demographic swamping, in which hybrids with reduced fitness decrease population growth rates, or (ii) genetic swamping, in which hybrids with moderate‐to‐high fitness replace pure wild genotypes over time (Todesco et al., 2016; Wolf, Takebayashi, & Rieseberg, 2001). It has long been assumed that crop–wild hybrids would have a lower fitness than their wild parent in natural habitats as has been shown, for instance, in sunflowers (Gutierrez, Cantamutto, & Poverene, 2011). However, in many other cases, hybrids showed no decrease in fitness, for instance in Sorghum (Arriola & Ellstrand, 1997). Some hybrids even display increased fitness compared to their parent species, for example in lettuce (Hooftman, Jong, Oostermeijer, & Den Nijs, 2007) and radishes (Hovick, Campbell, Snow, & Whitney, 2012). Assessing the threat posed by hybridization to the conservation status and future of wild species facing global changes thus requires not only estimating the degree of gene flow, but also evaluating the relative fitness of hybrids.

While crop‐to‐wild hybridization has been intensively studied in annual crops such as wheat (Arrigo et al., 2011) and rice (Pusadee, Schaal, Rerkasem, & Jamjod, 2013), its evolutionary consequence is still poorly known in perennial plant species such as fruit trees. Yet, tree species in general have high dispersal capacities and weak interspecific barriers (Petit & Hampe, 2006), two life history traits promoting hybridization such that wild tree populations can be threatened by introgression from cultivated trees. In this context, various conservation management methods are used, with advantages and drawbacks for each method. In situ approaches (i.e. conservation of natural populations in their native areas) are generally the preferred biodiversity management method for threatened forest trees, as they allow the continued evolution of populations and help maintain genetic diversity (Koskela et al., 2013). In situ conservation can also involve reintroducing individuals into areas from which the species has disappeared to create bridges among isolated populations. These individuals can be planted in hedges or in agroforestry programmes such as the ones carried out in France by the French Agroforestry Association (called hereafter *AFAF* for “*Association Française d'Agroforesterie*”). These conservation practices require the use of reliable pure and well‐identified wild seed source populations. The main issue is then to find reliable production networks for seeds, free from interspecific hybridization. One can harvest seeds from protected natural populations without introgressions. However, great effort is needed to find and harvest seeds from enough wild trees to avoid a loss of genetic diversity in the replanted populations. Alternatively, seed orchards, that is a dynamic ex situ conservation approach (Balsemin & Collin, 2004), allow harvesting large numbers of nonhybrid seeds easily. However, knowledge of pollen dispersal capacities and the factors affecting them is crucial for the creation of a seed orchard that would be isolated from crop gene flow to obtain seeds without introgressions.

One of the most widely cultivated fruit trees in the world is the apple tree *Malus domestica*. The genus *Malus* also includes ca. 30 wild species (Robinson, Harris, & Juniper, 2001), but its taxonomy is still unclear, in part due to weak interspecific barriers and also because outdated Latin names are still being used. For instance, *M. communis* is still used although it refers both to *M. domestica* and to crab apples. Several crab‐apple species occur in areas with widespread cultivation of apples, including *M. sylvestris* in Europe, an emblematic tree of European forests. One major threat for *M. sylvestris* is the disappearance of its preferred habitats, wood margins and hedges, in intensively cultivated areas. Another major threat is frequent introgression from the cultivated apple tree *M. domestica* (Coart, Van Glabeke, De Loose, Larsen, & Roldan‐Ruiz, 2006; Cornille, Gladieux, & Giraud, 2013; Gross, Henk, Forsline, Richards, & Volk, 2012; Larsen, Asmussen, Coart, Olrik, & Kjaer, 2006). Despite these threats, the conservation status of *M. sylvestris* is still unclear: while it is considered endangered in some European countries (e.g. Germany), the IUCN red list of threatened species indicates it as “data deficient” because the consequences of its hybridizations with *M. domestica* are still unknown (http://www.iucnredlist.org/). Such introgression is facilitated by a lack of interspecific reproductive barriers, by the self‐incompatible reproductive system that favours outcrossing and by the widespread cultivation of apples (Cornille et al., 2015). A previous study revealed high frequency of hybridization and crop‐to‐wild introgression in *M. sylvestris* populations (Cornille et al., 2015). An earlier study (Larsen et al., 2006), based on few F1 genotypes, suggested that hybrids may germinate earlier than pure *M. sylvestris* seeds. Studying the frequencies and consequences of *M. domestica‐*to*‐M. sylvestris* introgressions is particularly timely and critical in the light of the recent introduction of genetically modified apples, the “Arctic Apples” (Okanagan Specialty Fruits, Inc., www.fda.gov).

Our aim here was therefore to address the question of the conservation of the wild European crab apple in the face of introgression from *M. domestica*. More specifically, we wanted to (i) assess current hybridization rates, hybrid fitness and the suitability of current seed networks and (ii) provide recommendations for creating seed orchards protected from introgression, by investigating pollen dispersal ability. Two previous studies (Larsen & Kjaer, 2009; Reim et al., 2015) have reported pollen dispersal curves in European crab apples, but without studying dispersal in the light of hybridization or fitness differences between *M. sylvestris* and *M. domestica*. To investigate these issues, we chose the Dourdan forest situated 40 km south‐west of Paris (France) as our study area because it contains the single seed orchard of putative *M. sylvestris* trees from which the French National Forest Agency (*ONF* hereafter, “*Office National des Forêts*”) collects all *M. sylvestris* seeds that they use and supply to agroforestry programmes. We also analysed crab‐apple seeds sold by seed companies and seeds used by the *AFAF*, a French nongovernmental organization planting *M. sylvestris* trees, among other species, to reintroduce native trees into agrosystems.

We used microsatellite markers to genotype seeds from companies, adult trees and seeds from the Dourdan forest, and we grew seeds in a greenhouse, for addressing the following specific questions: (i) Are seeds sold and grown as crab‐apple seeds (from private companies, from the *ONF* seed orchard in the Dourdan forest and from the AFAF) free from introgression? (ii) What is the frequency of introgression of the crab apples from the Dourdan forest, both in adult trees and in seeds? (iii) What is the impact of the degree of *M. domestica* ancestry level on crab‐apple seed fitness? In particular, what is the impact of the degree of *M. domestica* ancestry level of the mother tree on the germination of its seeds? Further, what is the impact of the degree of *M*. *domestica* ancestry level of each seed on its germination ability, that is its germination speed and the growth rate of the resulting seedling? (iv) What are the pollen dispersal capacities of apple trees in a forest? Is this dispersal impacted by tree density and by the degree of *M. domestica* ancestry level? What are the pollen dispersal capacities of apple trees in the Dourdan seed orchard?

## 2 Materials and methods

### 2.1 Sampling

Our sampling site, the Dourdan forest and seed orchard, is situated in France, covering 14 km^2^. We collected leaves from 163 adult apple trees (mostly putative *M. sylvestris* but also a few *M. domestica* and putative hybrids) in the Dourdan forest and recorded their geographic coordinates (except for nine of them that were provided at a later stage by the Dourdan forest ranger). This sampling comprises all the apple trees known by the forest ranger in the area and therefore well represents the wild European crab‐apples population of this forest. We also collected leaves from the seed orchard in which *M. domestica* stocks were planted and grafted with branches of *M. sylvestris*. As some of these grafted trees had been insufficiently pruned, we were able to collect leaves from the stock (*N = *6) and grafted (*N *=* *14) branches. We also collected leaves from three stock trees on which the grafts did not take. Among the 186 sampled apple trees, 112 bore fruits in the fall 2013 and 2014 when we sampled. We collected the apples on the tree when possible or around the trunk where no confusion was possible regarding the mother tree. We collected all the fruits we could find and reach, up to 73 apples per tree. We measured the diameter of the apples and the mean number of seeds they contained.

We also collected leaf samples from trees planted by the AFAF between 2010 and 2015 (*N = *12) and from seedlings obtained from seeds sold by commercial seed companies (*N *=* *13). The French seed company *Vilmorin* sells seeds of *“M. communis,”* the Belgian *Sluyza* company sells seeds of *“M. communis (sylvestris)”* and *Forestart* in the United Kingdom sells seeds of *“M. sylvestris.”* The Dutch *Kuiper* company sells seeds whose names oddly associate the wild species name with that of *M. domestica* cultivars (*M. sylvestris* “A2,” *M. sylvestris* “Antonovka” and *M. sylvestris* “Bittenfelder”). We obtained a few samples from seedlings originating from three of these seed companies: *Vilmorin “M. communis”* (*N *=* *7), Sluyza *“M. communis (sylvestris)”* (*N *=* *2) and *Forestar*t *“M. sylvestris”* (*N = *4). We could not obtain samples from the Kuiper seed stocks.

### 2.2 Seed emergence and seedling growth rates

In a first experiment, for fitness analyses as a function of mother genotype, seeds were stratified in the dark for three months at 5°C in a damp mix of sand and vermiculite (ratio 2:1). Then, 30–60 seeds per mother (or all the seeds collected when fewer than 30 seeds were available, giving *N = *3,927 in total) were planted in soil in a greenhouse with controlled temperature (between 20 and 25°C) and constant day length (16 hr) and kept moist. Germination was checked two months after planting. For analysis of pollen dispersal distance, leaves from 311 seedlings were sampled to be genotyped (from 28 mother trees, 3 to 13 per mother, with a mean of 11 seedlings per mother).

In a second experiment, for fitness analyses as a function of both seed and mother genotypes, 344 seeds (7–14 seeds for 24 mothers) were stratified for two months at 5°C and were then moved in a greenhouse with controlled temperature (between 20 and 25°C) and constant day length (16 hr). Emergence of the seedlings was checked every 3 or 4 days, and the height of each seedling and number of leaves were recorded. When seedlings were more than 1.5 cm tall on the first day observed, we considered they had emerged the previous day. When the seedlings had more than seven leaves, we collected leaves for genotyping (*N *=* *138). After two months, we also retrieved as many nongerminated seeds from the soil as possible (*N *=* *72). The genotypes obtained through this second experiment were also used for the pollen dispersal distance analysis.

### 2.3 Genotyping

We extracted DNA from leaves from 186 adult trees, 12 samples from the *AFAF*, 13 samples from commercial seeds, 449 seedlings issued from seeds collected from the Dourdan forest and orchard and from 172 seeds (including 100 seeds coming from the orchard and 72 nongerminated seeds retrieved after the second experiment described above). Seeds were dissected to keep only the embryo. Both leaves and embryos were frozen at −80°C before grinding with metal beads. DNA was then extracted with the Nucleo Spin plant DNA extraction kit II^®^ (Macherey & Nagel). Multiplex microsatellite PCR amplifications were performed with a Multiplex PCR Kit^®^ (QIAGEN, Inc.). We used 33 microsatellite markers spread across the 17 chromosomes using 10 different multiplex reactions as previously described (Cornille et al., 2012). The genotyping was performed at the *Gentyane* platform (INRA). Only the microsatellite markers and individuals with <50% missing data were used for the rest of the analyses. The data set was then composed of the genotypes for 30 microsatellite markers from 186 adult samples from Dourdan, 12 samples from the *AFAF*, 13 samples from commercial seeds, 442 from the Dourdan forest and orchard, and 167 seeds (including 99 seeds from the orchard and 68 nongerminated seeds) retrieved after the second experiment described above. We used the software Cervus (Marshall, Slate, Kruuk, & Pemberton, 1998) to check for identical genotypes in the adult tree data set.

### 2.4 Paternity and dispersal analyses

Paternity analyses were performed on the seedlings of the greenhouse experiments (*N = *442), from the seeds from the orchard (*N = *99) and from the nongerminated seeds retrieved after the second experiment described above (*N = *68). We used Cervus (Marshall et al., 1998) with standard settings to match the genotype of offspring and mother tree in order to check both the identification of mother trees and the validity of markers. We only kept the microsatellite markers with <3% of incongruences between the genotypes of mother trees and their offspring. Any seed showing more than two mismatches from the genotypes expected given the mother's genotype were removed from the dispersal analyses. We thus retained 25 markers and 583 offspring genotypes. More than 80% of mismatches were found for homozygous loci and were thus probably due to null alleles (i.e. failed amplification of one allele). We then used Cervus to identify the father for each offspring using a paternity analysis. We used a two‐mismatch threshold between tree and seed genotypes and a three‐mismatch threshold when considering both parent trees and seed genotypes.

### 2.5 Hybridization analyses

In order to identify the hybrid status of the samples, we used reference genotypes from our collections previously identified as pure *M. domestica* (*N = *42) and pure *M. sylvestris* (*N = *31) (i.e. varieties assigned at a minimum of 90% to the appropriate genepool using STRUCTURE analyses, Cornille et al., 2012). In order to prevent any artefact in assignments due to population structure in *M. sylvestris* at the European scale, we used as references only *M. sylvestris* genotypes from regions belonging to the same genetic cluster as Dourdan (i.e. France and Belgium, Cornille et al., 2015).

For each genotype, we estimated a hybrid index with the add‐on R‐package *introgress* (Gompert & Buerkle, 2010). This hybrid index is an estimate of the proportion of alleles that were inherited from one of two hybridizing parental populations, that is the level of ancestry from one of two populations. In our case, pure *M. domestica* genotypes had a “hybrid index” of one while pure *M. sylvestris* genotypes had a hybrid index of zero. We therefore call this “hybrid index” *P*_*dom*_ as it represents the proportion of assignment to the *M. domestica* genepool.

### 2.6 Genetic diversity and statistical analyses

The allelic richness A_R_ was estimated with ADZE (Szpiech, Jakobsson, & Rosenberg, 2008), a software implementing a rarefaction method to analyse allelic diversity across different populations while correcting for sample size differences.

To compare genetic diversity (A_R_) between forest and orchard trees across markers, we used a signed‐rank test on the differences in this A_R_. To assess the impact of *M. domestica* ancestry level of the mother tree on its fitness and that of its progeny, several phenotypic traits related to fitness (apple diameter, number of seeds per fruit and germination rate) were regressed (linear regression) against the *P*_*dom*_ value of the mother. We averaged the measures for the duplicated genotypes of mother trees in the orchard (i.e. multiple grafting of the same clone), to avoid pseudo‐replication. We tested whether trees that did or did not bear fruit differed for their *P*_*dom*_ with a *t*‐test. To assess the impact of the *P*_*dom*_ of seeds on their germination ability (in the second experiment), we used a paired *t*‐test to compare the mean *P*_*dom*_ of germinated versus nongerminated seeds from each mother. To evaluate the impact of *M. domestica* ancestry level of the seeds on their fitness, we calculated the growth rate and the rate of increase in leaf number over the time period where these rates were linear (i.e. when they were not significantly nonlinear, which covered the period between days 2 and 29 after moving the seeds to the greenhouse for growth and between days 3 and 40 for leaf number). In order to avoid pseudo‐replication due to the presence of multiple half‐siblings (sharing their mother), we analysed the means of these traits per mother while including the sampling year as a blocking factor (which had no significant effect), retaining only the means performed on at least three germinated seeds. We then regressed germination timing, growth rate and rate of increase in leaf number against the *P*_*dom*_ of the seedlings. A mixed‐model multiple linear regression analysis, with the mother tree's identity as a random blocking factor, was run to investigate effects of tree density and *P*_*dom*_ of the parent trees on pollination distances. All of the statistical analyses were performed using the software JMP v7 (SAS Institute), using arcsine‐transformed values for *P*_*dom*_ and rates to improve normality.

To check for a correlation between geographic distances between trees and similarity in their introgression levels, we calculated the difference in P_*dom*_ for each pair of trees. We then used a Mantel test to compare the introgression difference matrix with that of the pairwise geographic distance using the R‐package *ncf* (Bjornstad, 2013).

## 3 Results

### 3.1 Hybridization levels

All samples from commercial seed companies that were sold as wild crab apples had substantial levels of *M. domestica* ancestry: 0.65 ≤ *P*_*dom*_ ≤ 0.98 for *Vilmorin*, 0.35 ≤ *P*_*dom*_ ≤ 0.36 for *Sluyza* and 0.76 ≤ *P*_*dom*_ ≤ 1 for *Forestart* samples. Of the 12 trees planted by the *AFAF* that we analysed, five could be considered as pure *M. sylvestris* (*P*_*dom*_ < 0.2) and two were assigned to *M. domestica* (*P*_*dom*_ > 0.8); the others had a mean *P*_*dom*_ of 0.3.

In the seed orchard, only 7% of the genotyped seeds had a *P*_*dom*_ > 0.2. The orchard was composed of stock trees on which *M. sylvestris* branches had been grafted. However, the trees had been insufficiently pruned, bearing ungrafted *M. domestica* branches with flowers. Of the 17 trees in the orchard, three no longer bore grafted *M. sylvestris* branches at all. Among the 14 orchard trees with grafted branches, only three different *M. sylvestris* genotypes were found. These three genotypes had low *M. domestica* ancestry (two with *P*_*dom*_ < 0.1 and the third with *P*_*dom *_= 0.16). Two trees with genotypes identical to those of the grafts were identified elsewhere in the forest, suggesting that these may have been two of the three graft‐donor *M. sylvestris* trees. The trees without grafted branches and the stock branches collected (*N *=* *6) had all the same, triploid *M. domestica* genotype.

The *M. sylvestris* genetic diversity was significantly lower in the orchard than in the forest, both for the adult trees (mean allelic richness across markers A_R _= 7.02 in the forest and A_R _= 4.06 in the orchards; *t *=* *13.09, *df*=24, *p *<* *.0001) and for the seeds (A_R _= 7.06 in the forest and A_R _= 3.96 in the orchards; *t *=* *9.96, *df *= 24, *p *<* *.0001).

**Figure 1:**
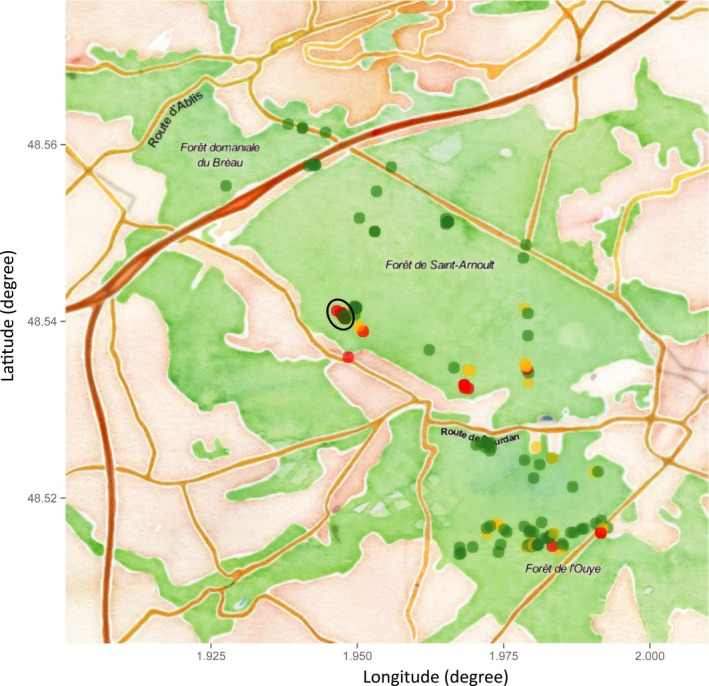
Spatial distribution of apple trees in the study area. Trees are represented by dots, and their colour indicates their degree of *Malus domestica* ancestry (*P*_*dom*_): green, *P*_*dom*_ < 0.2; red, *P*_*dom*_ > 0.8; and yellow, intermediate. The seed orchard is circled in black

**Figure 2:**
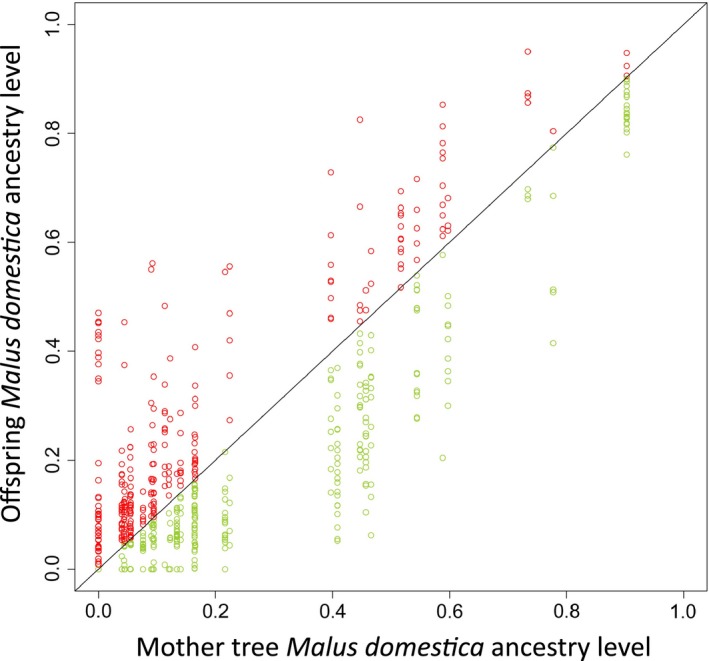
Level of *Malus domestica* ancestry in mother trees and in their progeny (602 progenies from 43 mothers sampled in the Dourdan forest and orchard). The line represents equal *M. domestica* ancestry between mother and seeds. Circles are coloured in red above the line and in green underneath

Estimates of the levels of *M. domestica* ancestry of the adult trees growing in the Dourdan forest (*N = *163) showed that 70% belonged to *M. sylvestris* (*P*_*dom*_ < 0.2), 7% to *M. domestica* (*P*_*dom*_ > 0.8) and 23% were hybrids (Figure 1). The *M. domestica* ancestry level of the seeds coming from *M. sylvestris* mothers (i.e. mothers with *P*_*dom*_ < 0.2) was significantly higher than that of their mothers in the forest, but not in the orchard (paired *t*‐test with the means per mother; in the forest: *t *=* *2.57, *df *= 16, *p *=* *.0032; in the orchard: *t *=* *0.30, *df *= 11, *p *=* *.6772; Figure 2).

### 3.2 Fitness of seeds as a function of hybridization level

In the first fitness experiment, we examined variation in fruit abundance and size, seed number and seed germination as a function of variation in hybridization level of mother trees. We detected no significant difference in mean *M. domestica* ancestry between trees with and without fruits (*P*_*dom*_ **= 0.24 and *P*_*dom*_ **= 0.23, respectively; *t *=* *0.19, *df *= 158, *p *=* *.8456). Trees with a higher level of *M. domestica* ancestry bore larger fruits (*N *=* *1,679 apples from 90 mothers, using mean fruit diameter from mothers with at least five measurable fruits, averaged over identical genotypes, *N *=* *77; *F*_(1,75)_ **= 62.43, slope ± SE* *= 1.63 ± 0.21; *r² *= 0.45; *p *<* *.0001). However, these larger fruits contained fewer seeds, the mean number of seeds per fruit per mother decreasing with the level of *M. domestica* ancestry (using mean seed number per fruit per genotype from the mothers with at least five collected apples; *N *=* *2,160 apples; 11,481 seeds from 99 mothers; 87 genotypes; *F*_(1,85)_ **= 11.79; slope ± SE* *= −2.03 ± 0.59; *r² *= 0.12; *p *=* *.0009). Overall, 62% of the seeds germinated and the success of seed germination increased slightly but significantly with increasing mother *M. domestica* ancestry level (*N *=* *3,836 seeds, from 101 mothers with more than five planted seeds, germination rates being averaged across identical genotypes, *N *=* *87; *F*_(1,85)_ **= 8.99; slope ± SE* *= 0.22 ± 0.07; *r* ^*2*^ **= 0.10; *p *=* *.0036). On average, the germination success was 69% for seeds from *M. domestica* trees (0.8 < *P*_*dom*_ < 1), 57% for seeds from *M. sylvestris* trees (0 < *P*_*dom*_ < 0.2) and 72% for seeds from hybrids (0.2 < *P*_*dom*_ < 0.8). Fitting a polynomial (degree 2) instead of a line did not improve the fit.

**Figure 3:**
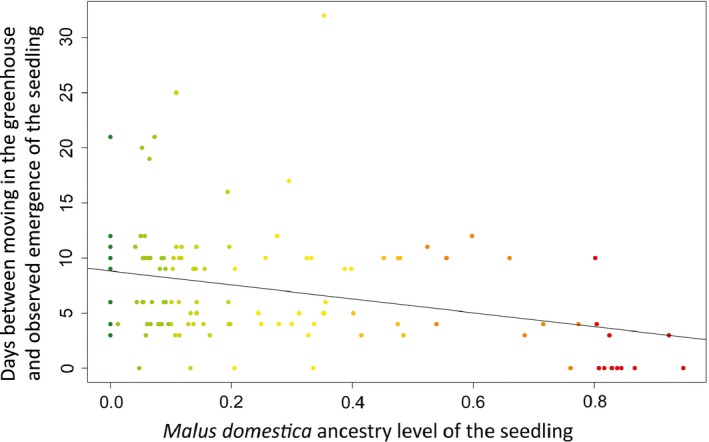
Days between moving the seeds to the greenhouse and observed emergence plotted against the level of *Malus domestica* ancestry of the seedlings (128 seedlings from 24 mothers sampled in the Dourdan forest and orchard). Colours indicate the level of *M. domestica* ancestry (red: *M. domestica*, green: *M. sylvestris* and shades of yellow/orange: hybrids). The line represents the linear regression

**Figure 4:**
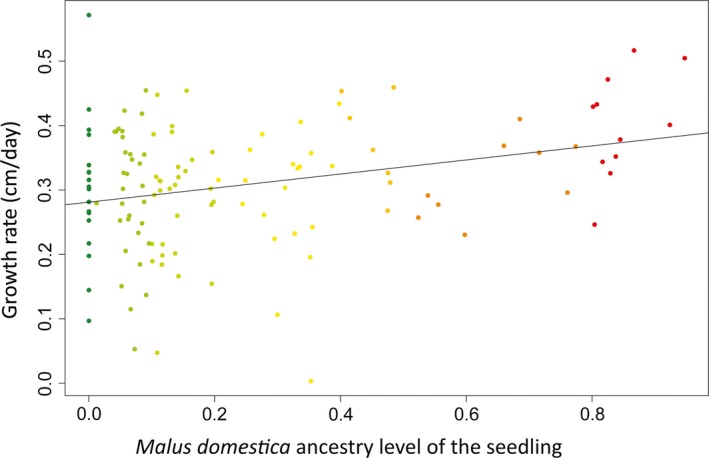
Growth rate of seedlings plotted against their level of *Malus domestica* ancestry (127 seedlings from 24 mothers sampled in the Dourdan forest and orchard). The colours indicate the level of *M. domestica* ancestry (red: *M. domestica*, green: *M. sylvestris* and shades of yellow/orange: hybrids). The line represents the linear regression

The second experiment was designed to check whether germination ability and growth were influenced by the hybridization levels of the seeds themselves, in addition to that of their mothers, by individually genotyping and monitoring seedling emergence, number of leaves and growth. We successfully genotyped 138 germinated seeds and 68 nongerminated seeds from 24 different mother trees. For the 22 mothers, for which we had both germinated and nongerminated seeds, a paired *t*‐test revealed no difference in their average hybridization levels (*t *=* *0.74, *df *= 21, *p *=* *.47). For the seedlings, we also recorded germination timing (i.e. how rapidly the seeds germinated), their rates of growth and of leaf number increase. Of the germinated seeds, maternal siblings with a higher *M. domestica* ancestry (*P*_*dom*_) emerged significantly earlier than those with a lower *P*_*dom*_ (*F*_(1,18)_ **= 5.28, slope ± SE* *= −4.88 ± 2.12, *r² *= 0.31, *p *=* *.0338, Figure 3) and gave rise to seedlings that grew more rapidly (*F*_(1,18)_ **= 5.42, slope ± SE* *= 0.11 ± 0.05, *r* ^*2*^ **= 0.25, *p *=* *.0318, Figure 4). In contrast, the rate of leaf appearance was not significantly correlated with *P*_*dom*_ (*F*_(1,18)_ **= 2.18, slope ± SE* *= −0.04 ± 0.02, *r² *= 0.11, *p *=* *.1619). We also checked for a correlation between heterozygosity and growth rate, rate of leaf number increase and germination timing (i.e. checking for heterosis or hybrid vigour), but we found no significant correlations (data not shown).

### 3.3 Pollen dispersal

A paternity analysis was performed on 583 offspring genotypes to identify adult tree genotypes compatible with the offspring genotype as possible pollen donors. We studied pollen dispersal and paternity separately for the forest and the seed orchard, because of its grafting history.

Among the 119 analysed offspring from the orchard, in no case was the putative male parent the *M. domestica* stock genotype. More than 90% of the seeds were sired by another of the three grafted *M. sylvestris* genotypes and only nine resulted from pollination events external to the orchard. For the four of these for which we could identify an unambiguous father in the forest, identified fathers stood from 132 to 167 m away from the mother tree.

For the forest seed samples, a male parent tree could be identified without ambiguity for 287 seeds from 30 mothers. For 26 seeds, several putative fathers were identified. We could identify no compatible father for 151 seeds. Half of these seeds came from only five mothers that had more than 80% of their offspring without identified fathers (three of them were located at the margins of our study area). Overall, we could only detect 10 seeds with genotypes consistent with self‐pollination events (i.e. 1.7% of the total number of seed genotypes analysed).

**Figure 5:**
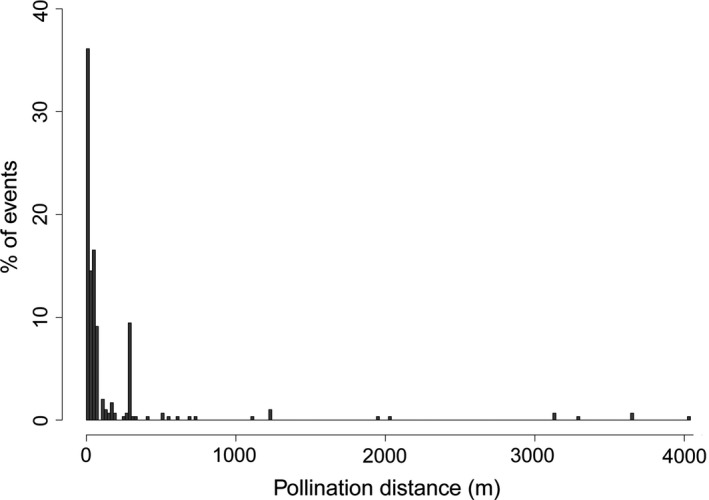
Distribution of the distance of pollination events (286 pollination events with a single putative father with known coordinates and from the 30 mothers in the forest) in the *Malus* trees of the Dourdan forest

The distance between the mother trees and the unambiguously detected male parent trees could be as large as 4 km (Figure 5). However, half of the pollination events for which we could identify the father occurred within <42 m, 25% at <15 m and 75% at <101 m. Altogether, although the vast majority of pollination events occurred at short distances, nearly 5% occurred between trees more than 1 km apart. A multiple regression analysis showed that pollination distances were significantly affected by apple tree density around parent trees (density being estimated as the number of apple trees within a radius of 100 m) and by the introgression level of the putative father (N = 286, *r* ^*2*^ **= 0.40, Table 1). Mothers in denser patches, as expected, had pollen donors that were nearer. The pollination distances increased with decreasing *M. domestica* ancestry in fathers.

**Table 1:** Mixed‐model multiple linear regression analysis investigating effects of tree density and degree of ancestry in *Malus domestica* (*P*_*dom*_) of the parent trees on pollination distances (286 pollination events with a single putative father with known coordinates and from the 30 mothers in the forest; ndf: numerator degrees of freedom; ddf: denominator degrees of freedom)

Effect	ndf, ddf	*F*‐ratio	*p*‐value
Tree density around mothers	1, 24.3	15.35	.0015
Tree density around fathers	1, 264.8	10.36	.0023
*Malus domestica* ancestry of mothers	1, 15.4	2.21	.1571
*Malus domestica* ancestry of fathers	1, 278.7	5.96	.0153

We also tested for assortative mating by level of *M. domestica* ancestry. The mean *P*_*dom*_ value of seed sires was significantly and positively correlated with that of the mother (test on the means per mother, for the 27 trees from the forest with more than three pollination events with an identified putative father: *F*_(1,25)_ **= 10.20, slope ± SE* *= 0.51 ± 0.16, *r* ^*2*^ **= 0.29; *p *=* *.0038). We checked whether this could result from spatial variation in introgression levels, that is if trees with similar levels of *M. domestica* ancestry were spatially clustered. We thus first calculated pairwise differences between levels of *M. domestica* ancestry among trees. This difference equals zero when two trees have similar levels of *M. domestica* ancestry and one when one is a pure *M. domestica* and the other a pure *M. sylvestris*. A Mantel test detected no significant correlation between pairwise geographic distance and this pairwise introgression difference index (*r *=* *−0.02, *p *=* *.1309), indicating a lack of spatial structure of introgression levels.

## 4 Discussion

Crop‐to‐wild hybridization and introgression are major threats to endangered wild populations. Historical crop‐to‐wild gene flow has been previously investigated in the European crab apple (Cornille et al., 2015). Here, we aimed at assessing contemporary interspecific gene flow as well as its fitness consequences for the wild crab apple. We also tested whether seeds sold or sampled as wild crab apples were free from introgression to provide recommendations for building orchards for growing *M. sylvestris* seeds, in particular by assessing pollen dispersal curves in forests with *M. sylvestris*,* M. domestica* and hybrid pollen.

### 4.1 Dispersal capacities are influenced by tree densities and introgression rates: consequences for future conservation programmes

The pollen dispersal curve was leptokurtic and fat‐tailed, that is with most pollination events occurring at short distances but with a substantial number of long‐distance pollination events. Here, almost 5% occurred farther than 1 km. The occurrence of relatively frequent long‐distance pollination events means that even *M. sylvestris* trees distant by several kilometres from any cultivated apple tree may still produce hybrid seeds. The pollen dispersal curve shape found here was consistent with the findings of previous studies on *M. sylvestris* (Larsen & Kjaer, 2009; Reim et al., 2015), but the dispersal distances varied: the maximum dispersal distance was around 300 m and 11 km in the two previous studies, respectively, and 4 km in our study. This variation may be explained by our finding that the pollen dispersal curve depends on tree density, as well as on the spatial scale of the study area.

Our results also showed that pollination distances decreased with the density of trees from the same species around the mother tree, which is in agreement with previous findings (Reim et al., 2015). Although this result is intuitive (pollinators fly shorter distances when there are more trees to forage from), it is of crucial importance from a conservation perspective. First, it highlights a further detrimental effect of habitat fragmentation on a species like *M. sylvestris*: more long‐distance pollination events potentially mean more pollination by *M. domestica* growing in gardens or orchards surrounding a forest or hedgerow. Second, this result helps in formulating guidelines for planting and maintaining seed orchards. High density of orchards will guard them against external pollen flow, effectively safeguarding the genetic pool of the seeds. In fact, in the orchard in the Dourdan forest, fewer than 10% of pollinations were external to the orchard itself and, of the cases for which the pollen donor could be identified, involved neighbouring trees.

We also found that the level of *M. domestica* ancestry influenced pollination in two ways. First, pollination distances decreased with the level of *M. domestica* ancestry of the father tree: the “wilder” trees had a higher pollen dispersal distance than more “domesticated” apple trees. Second, there was significant assortative mating regarding the level of *M. domestica* ancestry. Assortative mating is unlikely to be due to physical clumping of mother trees as we found no correlation between similarity in introgression level and physical distance. Assortative mating is most likely explained by temporal barriers to gene flow between cultivated and wild apples. Indeed, a previous study reported only partial overlap in flowering time between cultivated and wild apple trees, with peak flowering time differing by 1–2 weeks (Schnitzler, Arnold, Cornille, Bachmann, & Schnitzler, 2014).

### 4.2 A positive effect of introgression on fitness?

The question of the fitness of hybrids in crop–wild complexes is relevant from both basic and applied perspectives. The consequences of crop‐to‐wild introgression have been studied in different annual crops such as maize, rice or lettuce (Ellstrand et al., 2013); however, to our knowledge, this is the first time that such a study has been conducted on a perennial fruit tree. From a conservation perspective, it is important to know whether hybrids display lower or higher fitness than pure wild apple trees. In our first experiment, germination was higher for seeds from introgressed and *M. domestica* mothers although the effect was not significant in our second experiment, likely because the number of seeds was smaller and the overall germination rate lower (60% vs. 40%). Seeds with a larger *M. domestica* ancestry germinated earlier than wild seeds, as had been suggested in a previous study (Larsen et al., 2006), using only five *M. domestica* cultivars and five *M. sylvestris* genotypes. We also found that seedlings with a higher *M. domestica* ancestry grew faster than wild seeds. Overall, fitness at early life stages thus increased with the level of *M. domestica* ancestry. Although our experiments focused on the early developmental stages only and were performed in a greenhouse, these results suggest that hybrids probably suffer no reduced fitness in a natural environment either, at these stages. Therefore, hybrid seedlings can compete favourably with *M. sylvestris* seedlings where they occur naturally and may, over time, invade natural populations, if they display no disadvantages at later stages. This would explain the high levels of hybrids found here in the Dourdan forest and in a previous study all across Europe (Cornille et al., 2015).

### 4.3 Current crab‐apple seed network suffers from introgression, low genetic diversity and misidentified stocks

Our investigation of the French network providing *M. sylvestris* trees and seeds revealed several worrying aspects. First, there is an ambiguity about what species is sold, due to the use of an obsolete species name. Indeed, the name “*M. communis*” seems to be used either as a synonym for *M. sylvestris*, or as a way to name apple trees coming from uncertain ancestry or even for known cultivated variety names. *Vilmorin* does not explicitly sell their “*M. communis*” as a “wild” species but nor does it call them “*M. domestica*.” *Sluyza* also uses an ambiguous name, “*M. communis* (*sylvestris).” Kuiper* even uses “*M. sylvestris*” associated with the names “Antonovka” and “Bittenfelder” which are well‐known cultivated varieties. Every seed we could genotype coming from these companies had a *M. domestica* ancestry level of more than 0.3 (and very often more than 0.9), regardless of the name used. This means that buying genuine *M. sylvestris* seeds is extremely difficult if not impossible in the private sector. Indeed, identification of pure *M. sylvestris* individuals free from *M. domestica* pollination is quite difficult without genetic analyses, as attested by the high proportion of hybrids planted in agroforestry. In the French governmental *ONF* seed orchard, the trees from which the grafts were sourced belonged mostly to *M. sylvestris*. However, as we could only detect three distinct genotypes, the genetic pool of the orchard is very restricted, representing a very low sampling of the genetic diversity in *M. sylvestris*. This may lead to problems of inbreeding and poor adaptive potential if only seeds from this orchard are used to reintroduce the species into forests, as is the case currently.

## 5 Conclusion

Here, we have assessed the European crab‐apple pollen flow in the forest of Dourdan. Pollination distance decreased with increase in tree density and varied with *M. domestica* ancestry, with assortative mating probably due to some difference in flowering time between *M. sylvestris* and *M. domestica*. In addition, we have shown that substantial current crop‐to‐wild gene flow occurs in apple tree populations in natural habitats and that *M. domestica* trees and hybrids showed higher fitness than the wild apple tree at the early growth stages investigated. Overall, our results suggest increasing threats for wild apple integrity. Therefore, populations of European wild apples need to be protected by reducing interspecific gene flow and replanting genotypes free from introgressions. However, we have shown that the seed network feeding the programmes aiming at reintegrating the wild apple into forests and agrosystems suffers from poor genetic diversity and/or introgressions, and even misidentification of the seed species. These results altogether set a baseline for concrete conservation programmes of the wild apple. First, more *M. sylvestris* seed orchards should be set up, with high genetic diversity and protection from cultivated apple pollen, the latter being facilitated by planting stands of high local density far from cultivated apples. The trees used for producing source seeds for reintroduction schemes should be free of *M. domestica* introgressions, which can only be verified based on genotyping at several loci. A best first approach would be to take them from several forests previously identified as free from introgression (Cornille et al., 2015). However, care should also be taken to not mix too different genotypes, possibly locally adapted. In fact, a previous study has shown a strong population subdivision of *M. sylvestris* at the European scale (Cornille et al., 2015). These lineages should be kept separate in different orchards and replanted in their region of origin.

## Data Archiving Statement

Data available from the Dryad Digital Repository: http://dx.doi.org/10.5061/dryad.g12f9.
